# Preparation and Physicochemical Characterization of a Diclofenac Sodium-Dual Layer Polyvinyl Alcohol Patch

**DOI:** 10.3390/polym13152459

**Published:** 2021-07-27

**Authors:** Shafizah Sa’adon, Mohamed Nainar Mohamed Ansari, Saiful Izwan Abd Razak, Joseph Sahaya Anand, Nadirul Hasraf Mat Nayan, Al Emran Ismail, Muhammad Umar Aslam Khan, Adnan Haider

**Affiliations:** 1 BioInspired Device and Tissue Engineering Research Group, Faculty of Engineering, School of Biomedical Engineering and Health Sciences, Universiti Teknologi Malaysia, Skudai 81300, Johor, Malaysia; shafizahsaadon@gmail.com (S.S.); umar007khan@gmail.com (M.U.A.K.); 2 Institute of Power Engineering, Universiti Tenaga Nasional, Kajang 43000, Selangor, Malaysia; 3 Sustainable and Responsive Manufacturing Group, Faculty of Mechanical and Manufacturing Engineering Technology, Universiti Teknikal Malaysia Melaka, Hang Tuah Jaya, Malacca 76100, Malacca, Malaysia; anand@utem.edu.my; 4 Faculty of Engineering Technology, Universiti Tun Hussein Onn Malaysia, Batu Pahat 86400, Johor, Malaysia; nadirul@uthm.edu.my; 5 Faculty of Mechanical and Manufacturing Engineering, Universiti Tun Hussein Onn Malaysia, Batu Pahat 86400, Johor, Malaysia; emran@uthm.edu.my; 6 Institute of Personalized Medicine, School of Biomedical Engineering, Med-X Research Institute, Shanghai Jiao Tong University (SJTU),1954 Huashan Road, Shanghai 200030, China; 7 National Center for Physics, Nanoscience and Technology Department (NS & TD), Islamabad 44000, Pakistan; 8 Department of Biological Sciences, National University of Medical Sciences, Rawalpindi 46000, Pakistan; adnan_phd@outlook.com

**Keywords:** polyvinyl alcohol, diclofenac sodium, electrospinning, nanofiber, freeze-thaw process, cryogel

## Abstract

The aim of this study is to prepare a dual layer polyvinyl (PVA) patch using a combination of electrospinning techniques and cryogelation (freeze-thaw process) then subsequently to investigate the effect of freeze-thaw cycles, nanofiber thickness, and diclofenac sodium (DS) loading on the physicochemical and mechanical properties and formulation of dual layer PVA patches composed of electrospun PVA nanofibers and PVA cryogel. After the successful preparation of the dual layer PVA patch, the prepared patch was subjected to investigation to assess the effect of freeze-thaw cycles, nanofiber thickness and percentages of DS loading on the morphology, physiochemical and mechanical properties. Various spectroscopic techniques such as scanning electron microscopy (SEM), X-ray diffraction (XRD), Fourier transform infrared (FTIR), water contact angle, and tensile tests were used to evaluate the physicochemical and mechanical properties of prepared dual layer PVA patches. The morphological structures of the dual layer PVA patch demonstrated the effectiveness of both techniques. The effect of freeze-thaw cycles, nanofiber thickness, and DS percentage loading on the crystallinity of a dual layer PVA patch was investigated using XRD analysis. The presence of a distinct DS peak in the FTIR spectrum indicates the compatibility of DS in a dual layer PVA patch through in-situ loading. All prepared patches were considered highly hydrophilic because the data obtained was less than 90°. The increasing saturation of DS within the PVA matrix increases the tensile strength of prepared patches, however decreased its elasticity. Evidently, the increasing of electrospun PVA nanofibers thickness, freeze-thaw cycles, and the DS saturation has improved the physicochemical and mechanical properties of the DS medicated dual layer PVA patches, making them a promising biomaterial for transdermal drug delivery applications.

## 1. Introduction

Hydrogel patches are three-dimensional polymeric networks with a high-water content that disperse in water. Due to the presence of a unique cross-linked configuration, they are highly soluble in water. Hydrogels have a high degree of elasticity, variable biodegradability, a porous structure, biocompatibility, and tissue similarity, which means they will not react negatively with the body [1]. However, due to the hydrogels’ low mechanical strength and fragility, their use is still limited [2]. 

PVA is a semi-crystalline copolymer of vinyl acetate and vinyl alcohol that has found widespread use in biomedical and pharmaceutical applications due to its biocompatibility, non-toxicity, hydrophilicity, and nanofiber and hydrogel-forming capabilities. Despite its advantages, a previous study by Muppalaneni and Omidian [3] found that as the high-water content of PVA hydrogel shape began to expand, drug molecules will be solvated and practically leached out from the matrix, resulting in a relatively rapid release of drugs in a short period, particularly in the case of hydrophilic drugs. 

PVA hydrogels can be cross-linked in three ways: physically, chemically, or through radiation [4]. Physical crosslinking is the process of forming crystalline regions in a PVA solution by repeated freezing and thawing cycles; this results in the creation of a high strength, elastic gel. This procedure yields a physically cross-linked PVA hydrogel without the use of any chemical agents. Peppas et al. [5] used this process to produce PVA hydrogels with a higher strength and elasticity than room temperature PVA gels. One advantage of this technique is that no potentially hazardous reagents are retained after the gel is synthesized [6,7]. 

Freeze-thaw (cryogelation) is a technique for preparing hydrogels in which repeated freezing and thawing processes result in increased crystallization of PVA chains and the formation of a highly elastic cryogel. The degree of crystallinity is important in determining the rate of drug diffusion from cryogels, which can be used as a matrix or reservoir for drug delivery. The numbers of cycles are proportional to crystallization degree. With an increase in the number of cycles, the percentage of crystalline regions within the cryogel increases as well, resulting in a stiffer structure [8]. As the cryogel was frozen, the polymers formed ice crystals and became coarser as freezing times increased, resulting in the formation of the gel structure. Every time the ice was thawed (melted at room temperature), the cavities within the crystal structure were filled with polymers [7]. 

Electrospinning is one of the simple and effective fabrication methods for producing fibrillar structures made from a variety of biopolymers whose diameter can be formed from a nano to micro-scale. During the past several decades, electrospinning has progressed from single-fluid blending processes [9,10] to coaxial [11,12], tri-axial [13,14], side-by-side [15,16], and other complicated processes [17]. On the other hand, it is also combined with more and more traditional physical and chemical methods to expand its potential applications in the applied scientific fields [18,19]. The present work is an excellent example of the second approaches. Nanofibers produced by electrospinning exhibit several interesting and unique properties such as a controlled release carrier [20], high surface-to-volume ratio, tunable diameter and pore size, high porosity, surface functionality and morphology similar to the extracellular matrix; the use of electrospun nanofibers have been studied in diverse fields including drug delivery [21], wound dressing [22], filtration [23], cell culture, tissue engineering and many others [24,25]. Polymeric matrices such as PVA provide an excellent source for electrospinning based on their biocompatibility, extraordinary hydrophilicity, and mechanical properties [26,27]. However, according to Cui et al. [28], once electrospun PVA nanofibers are used as a drug delivery system, their morphology tends to be destroyed by the swelling due to continuous water absorption, and lead to a burst release of drugs due to their hydrophilic nature. Moreover, Ponrasu et al. [29] also reported the preparation of a fast-dissolving drug delivery system using PVA as a polymeric carrier, in which the drug was released rapidly from the nanofiber matrix. Some drug delivery systems are required to achieve sustained release, so it is necessary to modify the hydrophilicity of the nanofibers.

Diclofenac sodium (DS) is a commonly used non-steroidal anti-inflammatory drug that has been extensively used to treat a variety of conditions, including rheumatic disorders, arthritis, and soft tissue injuries [30]. DS is available in a variety of dosage forms, including oral and injectable forms for systemic dosing and topical products for local tissue treatment of underlying tissues [31]. Since oral administration of DS associates with low bioavailability and severe gastrointestinal side effects [32,33,34], transdermal delivery of DS hydrogel patches has been developed in recent years [35]. However, previous research has reported that DS loaded hydrogel patches encountered the problems of burst release, non-responsive drug release profile and heterogeneous drug distribution, since DS is partially soluble in water [36,37]. 

To the authors’ knowledge, the combination of electrospinning and cryogelation techniques for medicated patches have not yet been scrutinized. Therefore, a cost-effective dual layer PVA patch was prepared by combining electrospun nanofibers and cryogels of PVA to explore the stability of both layers with and without DS loading. Through this combined method, the related drawbacks of both systems (nanofibers and cryogel) can be overcome. 

## 2. Materials and Methods

### 2.1. Materials

Poly (vinyl alcohol) (PVA, molecular weight ~89,000–98,000 g/mol, 99+% hydrolyzed) was purchased from Sigma-Aldrich (St. Louis, MO, USA), with distilled water as a solvent. Diclofenac sodium (DS) ≥99.5% USP, molecular weight 318.13 gmol^−1^, was purchased from the Emory laboratory. Ethanol of approximately 95%, AR Grade, was purchased from QRëC^TM^ (Chonburi, Thailand). All other reagents were of analytical grade and used without further purification.

### 2.2. Methods

#### 2.2.1. Production of Electrospun PVA Nanofiber

**Figure 1:**
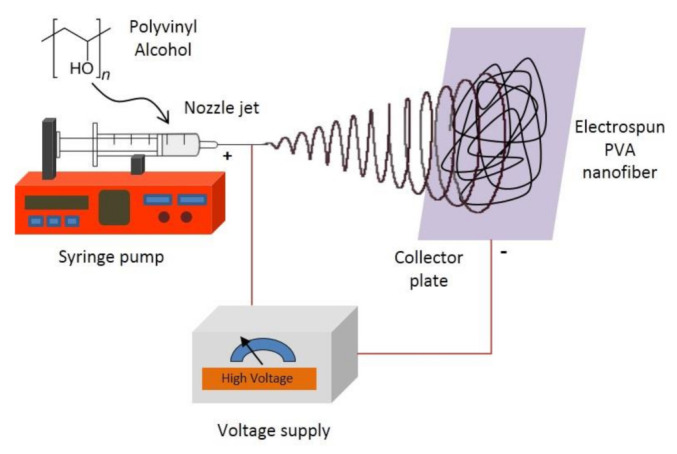
Electrospinning setup for electrospun polyvinyl alcohol (PVA) nanofiber production.

A weighed amount of PVA powder was dissolved in distilled water at 80 °C for 2 h to prepare a PVA solution at a fixed concentration of 10% *w/v*. After that, the solution was cooled down to room temperature (25 °C). Electrospinning of the freshly prepared PVA solutions was carried out by connecting the emitting electrode of positive polarity from a high voltage power supply model ES30PN/M692 by Gamma High Voltage Research (Florida, USA) to the solutions contained in a standard 5-mL syringe. The open end of this was attached to a blunt gauge-23 stainless steel needle (outer diameter = 0.91 mm), used as the nozzle, and the collection plate was laminated with aluminum foil (dimension = 15 cm × 15 cm), used as the fiber-collection device. A fixed electrical potential of 20 kV was applied across a fixed distance of 15 cm between the tip of the nozzle and the outer surface of the collector plate (i.e., the electrostatic field strength of (20 kV/15 cm). The feed rate of the solutions was controlled to about 1 mL/h utilizing a syringe pump. Figure 1 shows the schematic setup of the electrospinning process.

#### 2.2.2. Preparation of PVA Cryogel (Unmedicated and Medicated)

The aqueous PVA solution prior to the cryogelation process was prepared based on the procedure referred to in Section 2.2.1. The PVA was entirely dissolved and the obtained transparent solutions were slowly cooled to room temperature. The same methods were applied for DS loaded in PVA solution. A different mass% of DS (i.e., 1.0%, 1.5% and 2.0% *w/v*) was loaded by dissolving it in 5 mL ethanol in a separate beaker, and then adding it slowly to the above PVA solution by heating gently to avoid re-precipitation.

#### 2.2.3. Preparation of Dual Layer PVA Patch

The aqueous PVA solutions (from Section 2.2.2) were then poured on the surface of the electrospun PVA nanofibers that had been positioned on the specifically designed mold with dimensions: length × width × thickness: 8 cm × 8 cm × 1.5 cm. Cryogelation of the dual layer PVA patch was obtained by subjecting the PVA aqueous solutions to repeated freeze-thaw cycles (3 and 5 cycles), consisting of a 24 h freezing step at −20 °C followed by a 2 h thawing step at room temperature. The formulation of the dual layer PVA patch shown in Table 1. 

### 2.3. Characterization

#### 2.3.1. Scanning Electron Microscopy (SEM) of Electrospun PVA Nanofiber

Electrospinning was performed to prepared electrospun PVA nanofibers at room temperature. The morphological appearance of electrospun PVA nanofiber will be examined by using the SEM of JEOL-JSM6380LA (Tokyo, Japan), which operates at 15 kV at 30 µm magnification under a high vacuum. Each of the electrospun PVA nanofiber samples was sputtered with a thin layer of gold before SEM observation. Based on these SEM images, the average diameter of the PVA electrospun nanofiber been measured and reported as an average value. The width and diameter distribution of the nanofibers were determined by using Image J 1.44p Java 1.6.0_20 (32-bit) software with sampling sizes of at least 50 fibers from the SEM micrograph.

#### 2.3.2. Scanning Electron Microscopy (SEM) of Unmedicated and DS Medicated Dual Layer PVA Patch

To study the polymeric interaction between cryogels and nanofibers as well as the interaction of DS on prepared dual layer PVA patches, the micrograph of a dual layer PVA patch were performed. For this testing, the freeze-dried samples were cut to a small dimension, and directly sent to an Auto Fine Coater Machine for a sputtered thin layer of gold on its surface at 30 mA plasma current and 2 Pa of chamber pressure to make them conducting samples. The function of the coating is to make sure the insulating freeze-dried cryogel samples are electrically conductive during high-resolution electron imaging applications.

#### 2.3.3. X-ray Diffraction (XRD)

Wide-angle XRD profiles of dual layer PVA patches were obtained at room temperature using a Bruker AXS D8 Advance X-ray Diffractometer (Madison, USA), with a Cu-K α radiation source. The data was collected at 2*Ɵ* between 10°–70° degrees, wavelength of X-ray (1.54 Å). Samples were cut with dimensions 20 mm × 20 mm, and directly placed on the sample holder. 

#### 2.3.4. Fourier Transform Infrared (FTIR)

FTIR is used to investigate the interaction between the drug model and polymer of the dual layer PVA patch. Prepared dual layer PVA patch samples were cut into a small cube (10 mm × 10 mm × 10 mm) and placed at the FTIR sample holder. FTIR spectra were recorded in the range of 600 to 4000 cm^−1^ collecting 35 scans with 4 cm^−1^ resolution in the transmittance mode.

#### 2.3.5. Water Contact Angle

Wettability was estimated through water contact angle (WCA) measurement using a VCA Optima contact angle instrument (AST Products, Inc., Billerica, MA, USA) at room temperature. Further, 1.5 μL of distilled water had been dropped on the bottom-surface layer of the dual layer PVA patch.

#### 2.3.6. Tensile Test

Tensile strength, elongation at break and strain at break of unmedicated and DS medicated dual layer PVA patches with 1.0, 1.5 and 2.0 (% *w/v*) DS loading were measured by a Universal Testing Machine (LLOYD Instruments LR30K, Bognor Regis, United Kingdom). The crosshead speed used for the tensile test was 50 mm/min and the load range were 5 N. This specific dumbbell-shaped specimen (ASTM D412) had an overall length of 115 mm, with a gauge length of 33 mm long and gauge width of 6 mm.

## 3. Results and Discussion

### 3.1. Morphology Characterization

#### 3.1.1. Electrospun PVA Nanofibers

**Figure 2:**
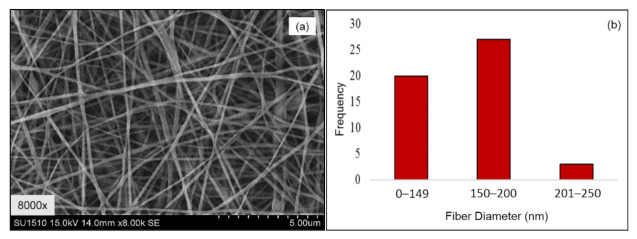
(**a**) SEM micrographs of PVA electrospun nanofibers with magnification 8000×; (**b**) average diameter of PVA electrospun nanofibers.

During electrospinning, as the liquid jet (polymeric solution) is continuously elongated, the solvent of the polymeric jet is evaporated quickly, phase separation occurs, the jet solidifies, and nanofibers are formed. The morphology and alignment of produced PVA electrospun nanofibers were examined using SEM. Figure 2a,b shows the micrographs of PVA electrospun nanofibers and average fiber diameters.

The morphological structure depicts no beaded nanofibers with diameters ranging from 90 nm to 250 nm (Figure 2b). The obtained PVA nanofiber had been further used to fabricate the dual layer PVA patch. The electrospinning process was performed as mentioned in Section 2.2.1. Table 2 shows the average thickness of the PVA electrospun nanofiber membrane for the 2- and 3-mL electrospinning running volume, which refers to A and B respectively.

From Table 2, data shows that the increasing volume of PVA solution in electrospinning has increased the thickness of the PVA electrospun nanofiber membrane. The spinning process with above-mentioned parameters produced non-uniform thickness of electrospun nanofibers. The thickness uniformity was taken at five different points. The average thickness for A electrospinning volume is 0.059 mm while the average thickness for B electrospinning volume is 0.086 mm.

#### 3.1.2. Prepared Dual Layer PVA Patch

**Figure 3:**
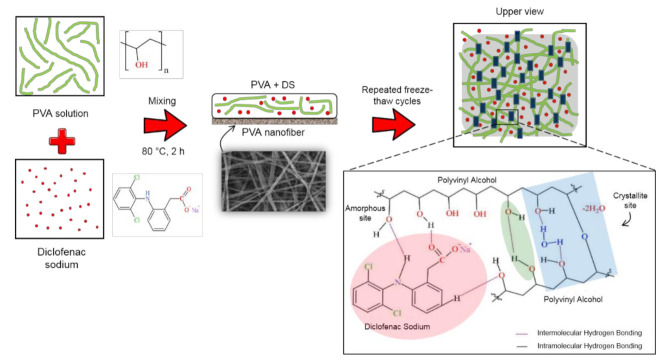
Cryogelation process of the DS medicated dual layer PVA patch.

The combined technique of electrospun nanofiber and freeze-thaw cryogel for preparation of unmedicated and DS medicated-dual layer PVA patches has been successfully conducted, as can be seen in Figure 3. The surface, bottom and cross-section of the dual layer PVA patch was immersed and cracked in liquid nitrogen, and then placed on the stub and coated with gold. The freeze–thaw cycles of PVA solutions determines the formation of the ice crystals into the amorphous region, which forces the polymer chains to arrange themselves into small, ordered regions (crystallites) [38]. The non-porous structure of prepared patches was also due to the shrinkage of the polymer network during freeze–thaw and freeze–dried processes, because of the influence of highly cross-linked PVA [39].

**Figure 4:**
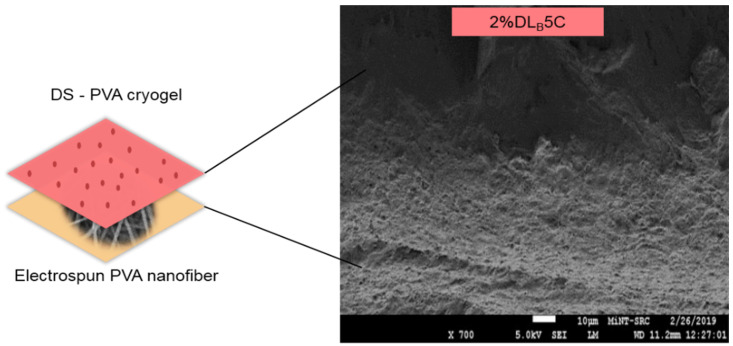
Cryogelation process of the DS medicated dual layer PVA patch.

The cross-section shown in Figure 4 confirmed the layer of the PVA cryogel (upper layer) and electrospun nanofiber (bottom layer) after finishing three and five freeze-thaw cycles. The purpose of this observation is to study the interaction between PVA electrospun nanofiber and PVA polymer after finishing each cycle. The cross-section of the dual layer PVA patch demonstrates the good polymer interactions between electrospun nanofiber and cryogel due to the hydrophilicity nature of the PVA. Even though the PVA is hydrophilic, the morphological structure of the PVA electrospun nanofiber still can be seen clearly under the SEM, although swollen in fiber diameter. This phenomenon might be due to the water absorption during the freeze–thaw process.

**Figure 5:**
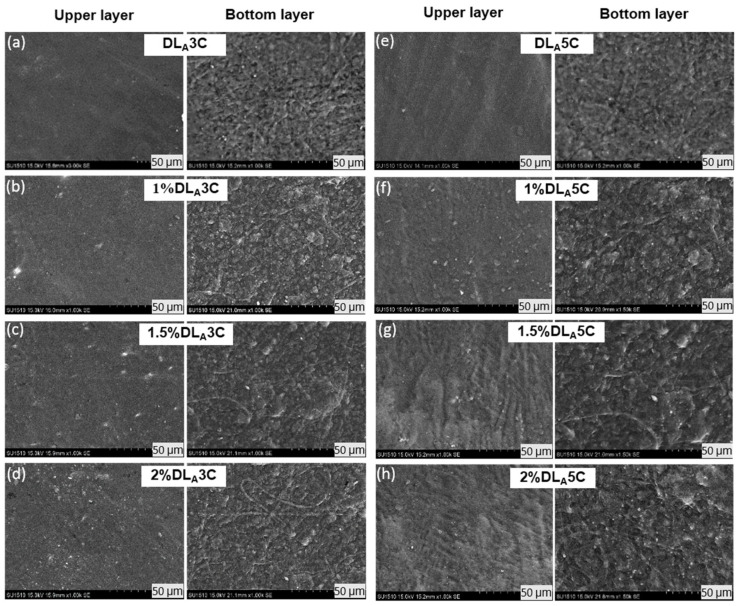
Morphological structure of unmedicated and DS medicated DL_A_3C (**a**–**d**) and DL_A_5C (**e**–**h**) patches.

**Figure 6:**
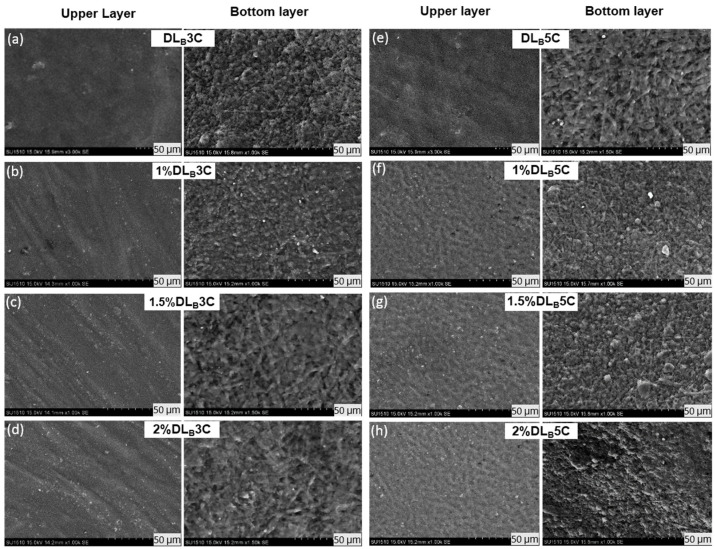
Morphological structure of unmedicated and DS medicated DL_B_3C (**a**–**d**) and DL_B_5C (**e**–**h**) patches.

Figure 5 and Figure 6 depict the surface and bottom layer of each composition to observe the variance of all prepared patches. Both Figure 5a–h and Figure 6a–h shows the morphological structures of the DL_A_ and DL_B_ for both cycles and all percentage of DS loading, respectively. From the microscopic observation, both figures show that the cryogel walls become denser and packed after five cycles, as evidenced by the PVA crystallites formation after a finished freeze–thaw process. These crystallites act as physical crosslinks. With repeated freeze–thaw cycles, the size and number of crystallites increased due to repetitive cycles causing further phase separation, forcing the water out of the liquid-like portions of the cryogel walls and increasing the concentration of local polymers [40,41]. 

The micrograph also reveals a rough surface of PVA cryogel after the freeze–thaw process is completed. This could be due to the presence of the DS molecules incorporated with PVA cryogel that interfered the intermolecular hydrogen bonding of PVA cryogel during the freeze–thaw cycle. DS is partly insoluble, so small particles of DS scattered on the surface of cryogel and nanofibers can be observed.

### 3.2. Effect of Nanofiber Thickness and Freeze-Thaw Cycle on Physico-Chemical Properties of Prepared Dual Layer PVA Patches

**Figure 7:**
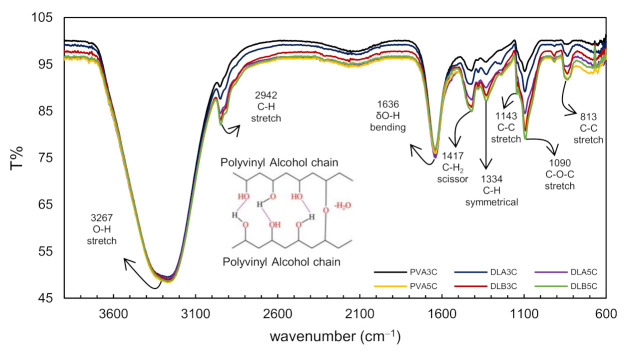
FTIR spectra of PVA cryogel and dual layer PVA patches with different PVA nanofiber thicknesses and freeze–thaw cycles.

**Figure 8:**
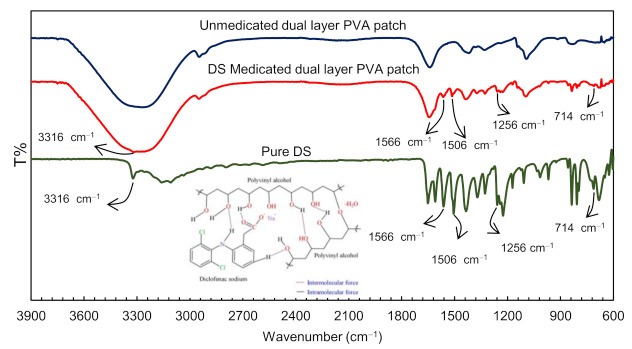
FTIR spectra of a pure DS unmedicated and a DS medicated dual layer PVA patch.

To explore the effect of all compositions of prepared patches towards the crystallinity of the PVA structure, the FTIR peaks of dual layer PVA patches are represented in Figure 7 and Figure 8. To strengthen the existence of the potential functional group in the dual layer PVA patch, the proposed chemical structures for both figures were displayed. Figure 7 shows the spectra of unmedicated PVA cryogel (PVA3C and PVA5C) and unmedicated dual layer PVA patches (DL_A_3C, DL_B_3C, DL_A_5C and DL_B_5C). 

In view of the results obtained in Figure 8, the characteristic absorption peaks of O–H stretching vibration from the inter- and intra-molecular hydrogen bonds were observed at 3267 cm^−1^, while C–H stretching vibration from alkyl groups was observed at 2942 cm^−1^ [42,43]. A strong band observed at 1634 cm^−1^ assigned to bending vibrations of the hydroxyl group corresponds to non-bonded water presence δ (O–H) [4]. 

Explicitly in Figure 7, the bending vibration is noticed at 1417 cm^−1^ and 1334 cm^−1^, which corresponds to a C–H_2_ scissoring and symmetrical bending vibration of C–H respectively [43]. Both 1143 cm^−1^ (C–C) and 1090 cm^−1^ (C–O–C) stretching vibrations observed are strongly correlated to the crystallinity of the PVA [44]. The vibration of aliphatic ether is associated with the stretching vibration, and this binding was possibly formed during physical crosslinking of polymer chains with a hydroxyl group (such as CH_2_–CHO–CH_2_~) that interacts with other hydroxyl groups of PVA polymer chains, forming bindings such as C–O–C by removing small molecules, such as water molecules (as shown in Figure 7) [45,46]. The peak around 913 cm^−1^ and 838 cm^−1^ tally to C–H_2_ rocking vibrations and C–C stretching vibrations, respectively [47].

Altogether, as can be seen in Figure 7, the peak intensities for the dual layer PVA patch increased as the freeze–thaw cycles and nanofiber thickness increased. These intensity patterns were possibly due to the increased volume of the functional group (per unit volume) correlated to the intermolecular or intramolecular interactions after the completion of the cryogelation process. 

To confirm the DS incorporation into the dual layer PVA patch, FTIR spectroscopy was employed on pure DS and DS medicated dual layer PVA patch (refer to Figure 8). Table 3 displays the tabulated spectra interpretation of pure DS. Based on data obtained, the FTIR spectra of prepared samples shows the characteristic peaks of the pure DS. This indicated the presence of intact DS on the prepared patch rather than being entrapped into the polymeric network. Theoretically, the entrapment of DS in the PVA polymeric network hindered the peak detection within the spectra [48]. 

As can be seen in Figure 8, the spectra exhibited a distinctive peak at 3316 cm^−1^ due to N–H stretching of the secondary amine. Meanwhile, peaks were observed at 1566 cm^–1^ and 1506 cm^–1^ owing to the –C=O stretch of the carboxyl group and C=C stretching of aromatic compound, respectively [49]. The IR peak also appearing at 1256 cm^–1^ resulted from C–N stretching of aromatic amine [50,51]. Peaks that can be assigned to the C–Cl stretching vibrations are visible in the region of 650–780 cm^−1^ [52]. 

These visible peaks can be attributed to the presence of DS in dual layer PVA patches, and show the increase in peak intensity as the percentage of the DS loading increased [53]. This is probably due to increasing frequencies of intermolecular forces between hydroxyl groups (-OH) in PVA polymer chains and DS structures (carboxyl group, organo-halogen compound group, aromatic amine) as shown in Figure 8. From the above interpretation, it is found that there is no shifting in the frequencies of the functional groups, proving that no DS–PVA interactions have been identified. Therefore, the result concludes that the DS was efficaciously loaded into the prepared patch. 

**Figure 9:**
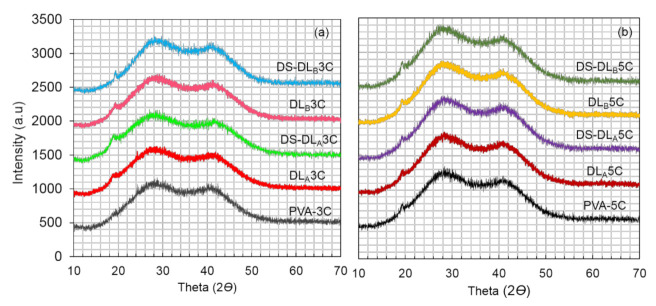
XRD pattern; PVA cryogel and unmedicated and DS medicated dual layer PVA patch (**a**) 3 cycles and (**b**) 5 cycles.

XRD analysis was conducted to understand the crystallographic effect of freeze–thaw cycles on the nanofiber layer and DS loading on the dual layer PVA patch discovered previously by FTIR. It is one of the most efficient, straightforward and convincing methods for the determination of material crystallinity, where the crystallite atoms absorb X-ray beams and subsequently diffract those beams, given specific directions [38]. It is imperative to know that crystallinity is one of the critical factors affecting the mechanical properties of a polymer. The pattern for PVA cryogel (PVA3C and PVA5C), in unmedicated (DL_A_3C, DL_B_3C, DL_A_5C, DL_B_5C) and DS medicated dual layer PVA patches (DS medicated-DL_A_3C, DS medicated-DL_B_3C, DS medicated-DL_A_5C and DS medicated-DL_B_5C), is presented in Figure 9a,b, respectively. 

**Figure 10:**
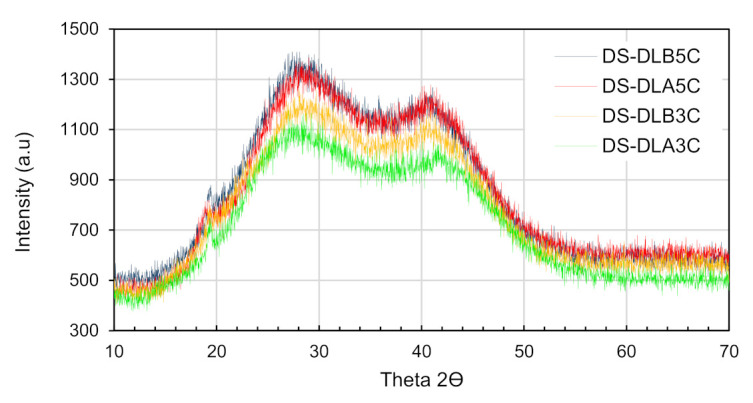
Comparison of XRD pattern of a DS medicated dual layer PVA patch for 3 and 5 cycles.

XRD profiles of PVA cryogel may be considered to be derived from the sum of three contributions: crystalline PVA aggregates, swollen amorphous PVA and free water. As shown in Figure 10, all samples revealed the same reflections with a low peak approximately at 2*Ɵ* ≈ 19.6°, corresponding to the (101) reflections plane of polymer crystalline phases [54,55]; the other two halos centered were found at 2*Ɵ* ≈ 28° and 41°, indicating the diffraction of free water in the PVA cryogel amorphous region as previously mentioned in the literature [56].

Referring to the XRD profiles, it is proven that the presence of a low amount of crystalline PVA aggregates due to interference from intermolecular hydrogen bonding among PVA chains during cryogelation process [57,58]. It can also be seen that the XRD pattern for dual layer PVA patches is increasing from DL_A_3C, followed by DL_B_3C, DL_A_5C and DL_B_5C. Additionally, the (101) crystal peak for a dual layer PVA patch was observed as being noticeably higher than PVA cryogel, proving that the combination of PVA nanofibers and PVA cryogel exhibits good polymer–polymer interactions with the dual layer PVA patch, as shown in Figure 4, simultaneously improving the crystalline phase. A previous study by Lee et al. [59] has revealed the enhancement of physical properties in PVA nanofibers by introducing a secondary crystallization through freeze–thaw processes, and suggested that the size of the crystalline phases in PVA nanofibers can be controlled up to five freeze–thaw cycles. 

Elucidating the effect of freeze-thaw cycles, PVA5C, DL_A_5C and DL_B_5C show marginally higher intensity compared PVA3C, DL_A_3C and DL_B_3C. This can be attributed to an increase of crystallite density upon finishing five freeze-thaw cycles, which led to the stable crystalline phase of the prepared patch as revealed in the FTIR spectra in Figure 7. It worth noting that the small increase of the (101) crystal peak is closely related to the rise in the number of well-packed regular PVA chains due to strong inter- and intra-molecular hydrogen bonding, as reported in the previous study [60,61]. 

A similar trend with a (101) crystal peak showed the intensity of the amorphous halos of the polymer at 2*Ɵ* ≈ 28, and 41° also shows a gradual increase from three to five freeze–thaw cycles. This may occur during the cryogelation process. Increasing freeze–thaw cycles induced the formation of ice crystals, causing elimination of water molecules in amorphous polymer segments, thus increased the polymer concentration in the swollen amorphous site [45]. 

As for the DS medicated dual layer PVA patch, no distinctive diffraction peak of DS crystallinity was detected in all samples. The absence of a DS crystallinity peak in the DS medicated-dual layer PVA patch infers that DS loading in the dual layer PVA patch is amorphous or molecularly dispersed within the polymer matrix, leading to a low sensitivity of drug crystalline detection [62,63]. In comparison, in Figure 9a,b, the results agreed that the intensity of a DS medicated dual layer PVA patch is slightly higher compared to an unmedicated dual layer PVA patch, due to the incorporation of DS molecules in the PVA polymer, which to some extent increased PVA crystallite density.

Figure 10 disclosed the crystallography effects of DS loading and freeze–thaw cycles on the dual layer PVA patch. It can be seen that the intensity of DS medicated DL_B_3C is noticeably higher compared to DS medicated DL_A_3C, while the intensity of the DS medicated DL_B_5C was almost as leveled as DS medicatedDL_A_5C observed. The same mechanism occurred with the different freeze–thaw cycles, where the intensity of DS medicated DL_B_5C and DS medicated DL_A_5C was considerably higher than DS medicated DL_B_3C and DS medicated DL_A_3C, respectively. 

These trends happen due to the increased crystal density of the prepared patch upon five freeze–thaw cycles which have restricted the drug mobility in the amorphous region, thus retaining the drug intact within the polymer. From overall results obtained, it can be understood that the incorporation of DS into the amorphous region of dual layer PVA patches somehow led to an increase in peak intensity due to the additional interaction of intermolecular hydrogen bonding between drug molecules and existing polymer concentrations. 

### 3.3. Wetting Properties of Prepared Dual Layer PVA Patch

**Figure 11:**
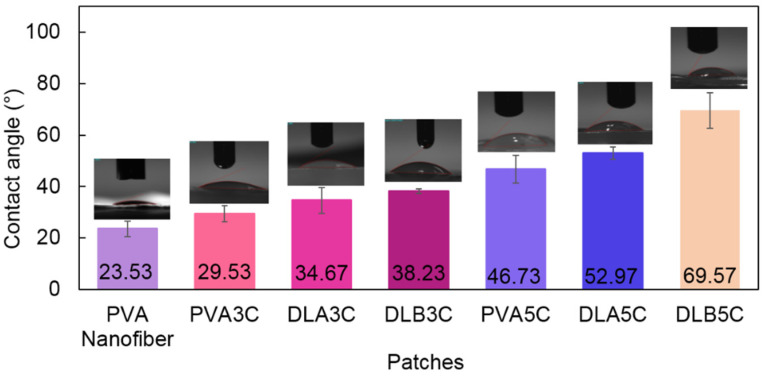
Wetting pattern of PVA nanofibers, PVA cryogel and a dual layer PVA patch (DL_A_ and DL_B_) for both cycles.

The wetting behavior of the surface of the dual layer PVA patch was investigated by the static angle with a contact angle meter by drop method. From the physical observation in Figure 11, the dual layer PVA patch acts as a hydrophilic patch. In all cases, their hydrophilicity decreases with the increase of freeze–thaw cycles and nanofiber thickness, which is expressed by the rise of water contact angles. 

As can be seen in Figure 11, the lowest degree of contact angle which can be observed is PVA nanofiber alone is at 23.53° followed by PVA cryogel with 29.53°, which nearly flattened as the PVA possessed good water solubility. On the other hand, DLB5C (60.57°) possessed a higher degree of contact angle followed by DLA5C (52.97°), DLB3C (38.23°) and DLA3C (34.67°). The presence of PVA electrospun nanofibers in DLB3C and DLB5C shows noticeable increases in the contact angle compared to DLA3C and DLA5C. This might be due to the polymeric interaction between PVA electrospun nanofiber and the cryogel of DLB, which is stronger than DLA as the layer of PVA nanofibers is thickening. It can be concluded that higher freeze–thaw cycles result in higher contact angles. 

This could be attributed to the increasing crystallite density as well as the stability of dual layer PVA patches after completing five cycles and corresponding to the roughness of PVA cryogel surfaces shown in the SEM images (Figure 5 and Figure 6). Nonetheless, although the trend of the contact angle is increased, the results indicate that the degree acquired was less than 90° for all prepared patches, which proves that the dual layer PVA patch is still considered to be highly hydrophilic, probably because of both intra- and inter-molecular hydrogen bonding involving the hydroxyl groups (–OH). These water contact angle results also determined that the hydrophilicity of dual layer PVA patches can be adjusted by adjusting nanofiber thickness and freeze–thaw cycles.

### 3.4. Tensile Properties of Unmedicated and DS Medicated-Dual Layer PVA Patches 

#### Effect of Nanofiber Thickness, Freeze–Thaw Cycles and Percentage DS Loading on Tensile Strength

**Figure 12:**
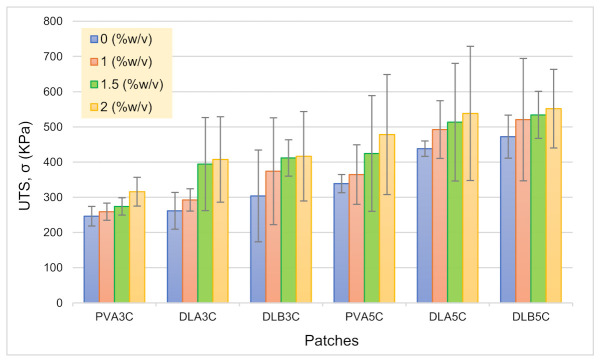
Ultimate tensile strength (UTS) of PVA cryogel, unmedicated and DS medicated patches.

**Figure 13:**
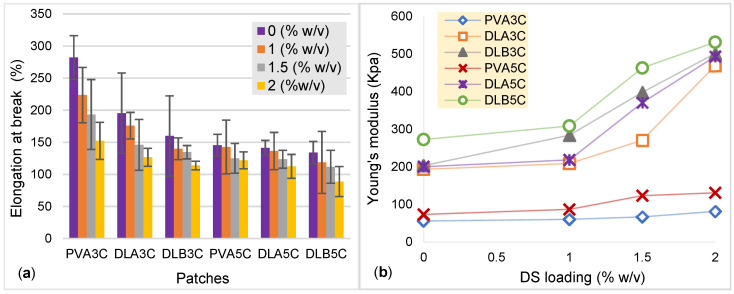
(**a**) elongation at break (%) and; (**b**) Young’s modulus (KPa) for PVA cryogel, unmedicated and DS medicated PVA patches.

In order to facilitate drug delivery, the patch should hold good mechanical properties to be used in a transdermal application. The effect of nanofiber thickness, freeze–thaw cycles and DS loading towards mechanical properties of PVA cryogel and dual layer PVA patches were studied. Both Figure 12 and Figure 13 show charts for the tensile strength, Young’s modulus and elongation at the break of PVA cryogel, unmedicated and DS medicated-dual layer PVA patches, respectively.

Based on results attained, the prepared patch displayed excellent tensile properties. As can be seen from the results, the dual layer PVA patch demonstrates higher tensile strength compared to PVA cryogel. It is known that the increase in the volume of electrospinning solution will increase the thickness of the nanofiber mats. It is evidently showing that the tensile strength of DL_B_ is higher than DL_A_ due to the thicker nanofibers attached to the PVA cryogel, resulting in a stronger dual layer PVA patch. Furthermore, when comparing prepared patches with both freeze–thaw cycles, results revealed that the prepared patch with five cycles showed notable tensile strength. This could be ascribed to the existence of larger sized crystallites contributing to the strength of the network as the number of freeze–thaw cycles is increased [64,65]. Such correlations clearly validate the role of freeze–thawing in obtaining cryogels with enhanced mechanical properties. Such findings are well in line with crystallinity principles. As crystallite density increased from three to five cycles, mechanical strength and modulus were also increase owing to the stability of the system, as explained in the above Section 3.2 and Section 3.3.

As can be seen in Figure 12, the incorporation of DS in prepared dual layer PVA patches also increased the tensile strength. The highest tensile strength and modulus observed is for the unmedicated dual layer PVA patch, and the DS medicated-dual layer is DL_B_5C and 2%DL_B_5C respectively, while the lowest for unmedicated and DS medicated-dual layer PVA patches is DL_A_3C and 1%DL_A_3C. The increasing drug content in PVA cryogel may decrease the crosslinking reaction, and consequently the gelation process is reduced significantly [7]. In view of the results obtained in Figure 13, the value of Young’s modulus is increased as the percentage of DS loading is increased. This indicates that the patch loses its elasticity as the drug molecule is deposited in-patch. In contrary to the percentage of elongation at break, it can be observed that, as the elasticity of the DS medicated-dual layer patch decreased due to the saturation of drugs in the polymer increasing, the prepared dual layer PVA patch has become stiffer and simultaneously decreases the percentage of elongation.

## 4. Conclusions

In this work, an unmedicated and DS medicated dual layer PVA patch was prepared using electrospinning and freeze–thaw methods in order to investigate the effect of freeze–thaw cycles, nanofiber thickness and percentage of DS loading on the physicochemical and mechanical properties of the prepared patch. This work was also devoted to assessing the stability of the incorporated DS in dual layer PVA patches in order to achieve the optimum loading percentage for transdermal drug delivery. The amount of percentage DS loaded was deliberately kept under 2.0% *w/v* to avoid saturation of the drug (DS) embedded in the dual layer PVA patches. It can be concluded that the dual layer PVA patch (unmedicated and DS medicated) using combined methods showed improvement in physicochemical and mechanical properties. The present work can further proceed with in vitro study to evaluate the pharmacokinetic profile of the DS medicated dual layer PVA patch.

## Figures and Tables

**Table 1:** Formulation of the diclofenac sodium (DS) medicated dual layer PVA patch.

Patches	Concentration of PVA (% *w/v*)	DS Loading (% *w/v*)	No. of Freeze-Thaw Cycles
* 2 mL-volume of electrospinning			
DL_A_3C	10	-	3
1%DL_A_3C		1.0	3
1.5%DL_A_3C		1.5	3
2%DL_A_3C		2.0	3
DL_A_5C		-	5
1%DL_A_5C		1.0	5
1.5%DL_A_5C		1.5	5
2%DL_A_5C		2.0	5
* 3 mL-volume of electrospinning			
DL_B_3C	10	-	3
1%DL_B_3C		1.0	3
1.5%DL_B_3C		1.5	3
2%DL_B_3C		2.0	3
DLB_5_C		-	5
1%DL_B_5C		1.0	5
1.5%DL_B_5C		1.5	5
2%DLB_5_C		2.0	5

* A = 2 mL electrospinning running volume; B = 3 mL electrospinning running volume.

**Table 2:** Formulation of the DS medicated dual layer PVA patch.

Electrospun PVA Nanofiber Mats	
A-Batch (mm)	B-Batch (mm)
0.059	0.085
0.042	0.086
0.069	0.093
0.064	0.080
0.061	0.086
0.059 ± 0.009	0.086 ± 0.004

**Table 3:** Tabulated FTIR interpretation of pure DS.

FTIR Interpretation of Diclofenac Sodium		
Sr. No.	Frequency (cm^−1^)	Characteristics
1	3316	N–H stretching of a secondary amine
2	1566	C=O stretching of carboxyl ion
3	1506	C=C stretching of an aromatic ring
4	1256	C–N stretching of aromatic amine
5	714	C–Cl stretching

## Data Availability

The data presented in this study are available on request from the corresponding author.
